# Omega-conotoxin MVIIA reduces neuropathic pain after spinal cord injury by inhibiting N-type voltage-dependent calcium channels on spinal dorsal horn

**DOI:** 10.3389/fnins.2024.1366829

**Published:** 2024-02-26

**Authors:** Nobuko Ohashi, Daisuke Uta, Masayuki Ohashi, Rintaro Hoshino, Hiroshi Baba

**Affiliations:** ^1^Division of Anesthesiology, Niigata University Graduate School of Medical and Dental Sciences, Niigata, Japan; ^2^Department of Applied Pharmacology, Faculty of Pharmaceutical Sciences, University of Toyama, Toyama, Japan; ^3^Division of Orthopedic Surgery, Department of Regenerative and Transplant Medicine, Niigata University Graduate School of Medical and Dental Sciences, Niigata, Japan

**Keywords:** omega-conotoxin MVIIA, neuropathic pain, spinal cord injury, N-type voltagedependent calcium channels, spinal dorsal horn

## Abstract

Spinal cord injury (SCI) leads to the development of neuropathic pain. Although a multitude of pathological processes contribute to SCI-induced pain, excessive intracellular calcium accumulation and voltage-gated calcium-channel upregulation play critical roles in SCI-induced pain. However, the role of calcium-channel blockers in SCI-induced pain is unknown. Omega-conotoxin MVIIA (MVIIA) is a calcium-channel blocker that selectively inhibits N-type voltage-dependent calcium channels and demonstrates neuroprotective effects. Therefore, we investigated spinal analgesic actions and cellular mechanisms underlying the analgesic effects of MVIIA in SCI. We used SCI-induced pain model rats and conducted behavioral tests, immunohistochemical analyses, and electrophysiological experiments (*in vitro* whole-cell patch-clamp recording and *in vivo* extracellular recording). A behavior study suggested intrathecal MVIIA administration in the acute phase after SCI induced analgesia for mechanical allodynia. Immunohistochemical experiments and *in vivo* extracellular recordings suggested that MVIIA induces analgesia in SCI-induced pain by directly inhibiting neuronal activity in the superficial spinal dorsal horn. *In vitro* whole-cell patch-clamp recording showed that MVIIA inhibits presynaptic N-type voltage-dependent calcium channels expressed on primary afferent Aδ-and C-fiber terminals and suppresses the presynaptic glutamate release from substantia gelatinosa in the spinal dorsal horn. In conclusion, MVIIA administration in the acute phase after SCI may induce analgesia in SCI-induced pain by inhibiting N-type voltage-dependent calcium channels on Aδ-and C-fiber terminals in the spinal dorsal horn, resulting in decreased neuronal excitability enhanced by SCI-induced pain.

## 1 Introduction

Spinal cord injury (SCI) is a serious event that can have devastating physical and mental effects on patients. The primary effect of SCI is the loss of motor function; however, the development of neuropathic pain is a common consequence prevalent in more than half of individuals with SCI. These patients experience severe pain, influencing their quality of life (Mariano, 1992; Yezierski, 1996; Siddall et al., 1999; Hulsebosch, 2002; Siddall et al., 2003; Ackery et al., 2004; Rossignol et al., 2007; Oyinbo, 2011; Finnerup, 2013; Finnerup, 2017; Burke et al., 2019; Widerström-Noga et al., 2023). At the time of injury, the primary lesion disrupts axons and neurons, interrupting nerve impulses and causing neurodegenerative events that worsen the initial injury. Several anatomical and neurochemical events occur in the spinal cord, including necrosis, apoptosis, oxidative stress, excitotoxicity, and neuronal tissue degeneration, possibly leading to neuropathic pain following SCI (SCI-induced pain) (Choi, 1992; Isaac and Pejic, 1995; Yezierski, 1996; Amar and Levy, 1999; Schumacher et al., 1999; Carlson and Gorden, 2002; Hall and Springer, 2004; Kwon et al., 2004; Aslan et al., 2009; Torres et al., 2010; Liu et al., 2011; Oyinbo, 2011; Hall et al., 2016; Xia et al., 2017). In SCI-induced pain, below-level neuropathic pain is particularly characterized by allodynia and hyperalgesia. These secondary mechanisms of injury are caused by complex factors, such as ionic disturbances, increased neurotransmitter release (glutamate), and inflammatory responses (accumulation of arachidonic acid and free radicals). Considering ionic disturbances, excessive intracellular calcium accumulation and voltage-gated calcium-channel upregulation play critical roles in secondary injury mechanisms of SCI-induced pain (Choi, 1992; Isaac and Pejic, 1995; Amar and Levy, 1999; Schumacher et al., 1999; Aslan et al., 2009; Torres et al., 2010; Liu et al., 2011; Oyinbo, 2011). Since the initial impact of injury can only be prevented, therapeutic strategies for SCI target the cascade of secondary events triggered soon after SCI. Thus, the development of novel neuroprotective approaches to SCI is critically dependent on understanding the calcium-mediated mechanisms underlying SCI (Choi, 1992; Isaac and Pejic, 1995; Amar and Levy, 1999; Schumacher et al., 1999; Aslan et al., 2009; Torres et al., 2010; Liu et al., 2011; Oyinbo, 2011). Therefore, calcium-channel blockers can improve SCI by preventing the intense influx of calcium ions and the progression of secondary injury (Choi, 1992; Isaac and Pejic, 1995; Amar and Levy, 1999; Aslan et al., 2009; Torres et al., 2010; Liu et al., 2011). Indeed, gabapentin and pregabalin, which are the first-line treatments for SCI-induced pain, act via binding to a G protein-coupled voltage-gated calcium channel α_2_δ subunit and presumably inhibit calcium channel (Baastrup and Finnerup, 2008; Finnerup and Baastrup, 2012). However, the role of calcium-channel blockers in SCI-induced pain remains unknown.

Many natural calcium-channel blockers have been identified, with neuroactive or neuroprotective peptides derived from different venomous species, such as toxins from the genus *Conus*, also called conotoxins. Omega-conotoxin MVIIA (hereafter designated MVIIA) is a calcium-channel blocker obtained from the marine snail *Conus magus* that selectively inhibits N-type voltage-dependent calcium channels (Valentino et al., 1993; Buchan et al., 1994; Hovda et al., 1994; Bowersox et al., 1997; Lewis et al., 2000; Verweij et al., 2000; Lewis et al., 2012; Sanford, 2013). Calcium-channel blockers, especially N-type voltage-dependent calcium-channel blockers, inhibit and modulate the release of various neurotransmitters, such as norepinephrine and glutamate (Gaur et al., 1994; Olivera et al., 1994; Bowersox et al., 1995; Ghosh and Greenberg, 1995; Igelmund et al., 1996; Gonçaves et al., 2011); however, MVIIA, among calcium-channel blockers, is the only analgesic drug administered via intrathecal (i.t.) injection that is clinically approved by United States Food and Drug Administration for severe chronic pain (Bingham et al., 2010; Lewis et al., 2012; Sanford, 2013; McDowell and Pope, 2016). MVIIA elicits neuroprotection against ischemia and traumatic brain injury (Valentino et al., 1993; Buchan et al., 1994; Hovda et al., 1994; Bowersox et al., 1997; Perez-Pinzon et al., 1997; Verweij et al., 1997; Burns et al., 1999; Berman et al., 2000; Verweij et al., 2000). Thus, MVIIA may contribute to the prevention of secondary spinal injury and may demonstrate neuroprotective effects. Although a recent human clinical study using ziconotide, N-type voltage-dependent calcium-channel blockers: MVIIA, in patients with SCI-induced pain reported a decrease in pain (Brinzeu et al., 2019), the effects and mechanisms of MVIIA on SCI-induced pain are still fully unknown. For example, a behavioral study using animal models investigated the effect of MVIIA administration 3 weeks after SCI on neuropathic pain (Hama and Sagen, 2009), while another study investigated the cell viability, including mitochondrial viability or cell death value (Oliveira et al., 2018). Therefore, the direct spinal analgesic effect and spinal cellular mechanisms of MVIIA were not investigated, especially when administered in SCI-induced pain. Therefore, we investigated whether MVIIA induces analgesia in SCI-induced pain model rats using behavioral tests. Furthermore, we focused on MVIIA neuronal mechanisms in spinal analgesia and assessed synaptic transmission in the spinal dorsal horn using immunohistochemical analyses and electrophysiological experiments (*in vitro* whole-cell patch-clamp recording and *in vivo* extracellular recording).

## 2 Materials and methods

### 2.1 Animals

This study was approved by the Institutional Animal Care and Use Committee of the Niigata University Graduate School of Medical and Dental Science (approval no. SA01015), and all experiments were performed in accordance with relevant guidelines and regulations. All animal procedures were conducted in accordance with ARRIVE guidelines and our previous animal studies (Ohashi et al., 2017, 2022); we minimized pain or discomfort to animals. Male Wistar rats (200–250 g) were used; the animals were housed with *ad libitum* access to food and water under a 12-h light/dark cycle.

### 2.2 SCI procedure

All surgeries were performed by the same experienced investigator, and contusion surgery was performed as described previously (Boroujerdi et al., 2011; Wu et al., 2013; Kusuyama et al., 2018; Sliwinski et al., 2018). Anesthesia was induced and maintained using isoflurane. The thoracic to the lumbar levels were shaved using an electric shaver. The rats were positioned in a stereotaxic apparatus. A midline incision was made in the T8–T12 region, followed by blunt dissection to expose the T10 region of the spinal cord. The spinous process of the T10 vertebra was removed via laminectomy, exposing the corresponding spinal cord region. A contusion injury was induced using an IH impactor device (Precision Systems and Instrumentation, LLC, Fairfax, VA, United States) that emulates a 100-kdyn weight-drop onto the dura mater at a distance of 3–4 mm. After the contusion injury, the connective tissue, muscle, and skin were closed with 5–0 chromic gut, and manual expression of the bladder was performed twice daily until the animals recovered the ability to void spontaneously.

### 2.3 von Frey test

To assess the effect of MVIIA on analgesia in SCI-induced pain, we conducted behavioral tests using the von Frey test as reported previously (Ohashi et al., 2017, 2022). The rats were acclimated to the experimental room at least 30 min before starting the experiments. To investigate sensitivity to mechanical stimulation and determine the force threshold of paw withdrawal, we applied each von Frey filament to the plantar surface of the hind paws (Ohashi et al., 2017, 2022). The withdrawal threshold was defined as the lowest force that evoked a clear withdrawal response at least twice in 10 applications. Observations were recorded before the contusion injury (control) and 14 days after contusion injury (day 14) and were evaluated by two blinded examiners. We decided to perform the von Frey test 14 days after the contusion injury to ensure sufficient motor function recovery.

### 2.4 Assessment of locomotor functions

To assess the neuroprotective effects of MVIIA, we investigated its analgesic effect and effect on motor function recovery in SCI. Locomotor function recovery after spinal contusion injury was monitored using the Basso, Beattie, and Bresnahan (BBB) scoring scale, which is based on the movement of the three hind limb joints (hip, knee, and ankle) (Basso et al., 1995, 1996). The rats were acclimated to their testing environment by handling and exposing them to an open-field test apparatus 60 min before surgery. Locomotor recovery was assessed on a scale ranging from 0 (no movement) to 21 (normal functioning) using the BBB scoring scale (Basso et al., 1995, 1996). The lower segment of the scale (0–7) measures the early recovery phase, which consists of isolated joint movements of the hip, knee, and ankle. The middle portion of the scale (8–12) represents the intermediate recovery phase, which corresponds to the plantar placement of the hind paw with or without weight support and plantar stepping. The upper portion of the scale (14–21) evaluates the late recovery phase and is assessed after the animal demonstrates consistent coordination between the front and hind limbs. This portion of the scale focuses on the paw position, toe clearance, trunk stability, and tail position. Observations were recorded for 10 min each day after the contusion injury (day 0) until 14 days (day 14) and were evaluated by two blinded examiners.

### 2.5 Immunohistochemical staining for phosphorylated extracellular signal-regulated kinase (pERK) in the spinal dorsal horn

To assess the activation of pERK, a marker of dorsal horn neuron activation and an indicator of pain, we performed immunohistochemical analyses, as reported previously (Ohashi et al., 2022). Fourteen days after contusion injury (day 14), rats were anesthetized using urethane (1.5 g/kg, i.p.), and dorsal laminectomy was performed. The lumbosacral segment of the spinal cord was removed, and the isolated spinal cord was placed in preoxygenated ice-cold Krebs solution. All ventral and dorsal roots, except the L5 dorsal root, were severed, and the arachnoid membrane was removed. The spinal cord was mounted on the stage of a microslicer (Linear Slicer PRO 7; Dosaka, Kyoto, Japan) and cut as a 650-μm-thick transverse slice attached to the L5 dorsal root. This transverse slice was transferred to a recording chamber and perfused with Krebs solution (10–15 mL/min), which was equilibrated with a 95% O_2_/5% CO_2_ gas mixture at 36°C ± 1°C for at least 3 h. The Krebs solution contained the following (in mM): NaCl, 117; KCl, 3.6; CaCl_2_, 2.5; MgCl_2_, 1.2; NaH_2_PO_4_, 1.2; NaHCO_3_, 25; and D-glucose, 11.5. Subsequently, the slice was fixed in 4% paraformaldehyde for 60 min, equilibrated with sucrose overnight, and cut into 30-μm-thick sections using a cryostat. Sections were rinsed in phosphate-buffered saline (PBS) and permeabilized in a solution of 0.3% Triton X-100 (Sigma Chemicals, St. Louis, MO, United States) in PBS (PBS-TX). Nonspecific binding was blocked with 10% normal goat serum (Vector Laboratories, Inc., Burlingame, CA, USA), diluted in PBS, and incubated for 1 h at room temperature. The sections were incubated with rabbit anti-pERK1/2 antibody (Cell Signaling Technology, Danvers, MA, USA; 1:1,000) in PBS-TX overnight at room temperature. After rinsing with PBS, the sections were incubated for 1 h at room temperature with biotinylated goat anti-rabbit antibodies. Immunoglobulin G (Vector Laboratories, Inc.) was diluted in PBS. The tissue sections were then rinsed in PBS and incubated for 1 h at room temperature with a Vectastain ABC system kit (Vector Laboratories, Inc.), following the manufacturer’s instructions. After further rinsing with PBS, the tissues were incubated in 3,3′ diaminobenzidine chromogen until a precipitate was visible on pERK-positive sites. A final rinse with water was performed to stop the chromogenic reaction. Tissue sections were slide-mounted, dehydrated, and covered with DPX non-aqueous mounting medium (Sigma-Aldrich, St. Louis, MO, United States). To count the number of pERK-positive neurons in the superficial spinal dorsal horn (laminae I–II) as described previously (Ohashi et al., 2017, 2022), at least five non-adjacent sections were randomly selected, and the neurons were counted under a microscope equipped with a digital camera system (Nikon, Tokyo, Japan).

### 2.6 *In vivo* extracellular recording from the superficial spinal dorsal horn

*In vivo* extracellular recordings from superficial spinal dorsal horn neurons were obtained, as described previously (Andoh et al., 2017; Uta et al., 2019, 2021). Fourteen days after contusion injury (day 14), the rats were anesthetized using urethane (1.5 g/kg, i.p.), and thoracolumbar laminectomy was performed to expose the spinal column from L3 to L5. The rats were then placed in a stereotaxic apparatus. After removing the arachnoid membrane to create a window large enough for a tungsten microelectrode, the surface of the spinal cord was equilibrated with Krebs solution (10–15 mL/min) and irrigated with a 95% O_2_/5% CO_2_ gas mixture at 36°C ± 1°C. The Krebs solution was the same as that described in the *Immunohistochemical staining* section. Extracellular single-unit recordings from lamina I-II neurons of the superficial spinal dorsal horn were obtained, and spikes were selected based on amplitude discrimination, according to previous studies (Andoh et al., 2017; Uta et al., 2019, 2021). The tungsten microelectrode (tip diameter, 25 μm; tip impedance, 9–12 MΩ) was inserted into the spinal dorsal horn of the ipsilateral side at an angle of 20 °–30 ° (latero-medial), and recordings were obtained 20–100 μm below the surface, corresponding to lamina I-II neurons of the superficial spinal dorsal horn. The signals were amplified, digitized, and displayed online using the pCLAMP 10.2 software suite (Molecular Devices, Union City, CA, USA). We searched for the region on the skin where neural responses were produced by touching with a cotton wisp and/or light brush or a noxious pinch with forceps. Neurons were classified as the wide dynamic-range type if they responded to both an innocuous mechanical stimulus and a noxious pinch or as the nociceptive-specific type if they responded to a noxious pinch but not to a cotton wisp or brush stimuli. Using a von Frey filament with a bending force of 8 g, mechanical stimulation was applied to the caudal dorsal skin of the ipsilateral side for 10 s (Akiyama et al., 2012). For quantification, the spontaneous firing rate before stimulation was subtracted from firing rates evoked by the von Frey filament.

### 2.7 *In vitro* patch-clamp recordings from substantia gelatinosa neurons in lamina II

*In vitro* patch-clamp recordings from substantia gelatinosa neurons were performed as described previously (Ohashi et al., 2017, 2022). Transverse spinal cord slices from the rats 14 days after contusion injury (day 14) were prepared as described in the *Immunohistochemical staining* section, and MVIIA was bath-applied directly to these transverse spinal cord slices during the recordings. Under a dissection microscope with transmission illumination, lamina II of the dorsal horn was discernible as a relatively translucent band across the dorsal horn. Whole-cell patch-clamp recordings of substantia gelatinosa neurons in lamina II were obtained in the voltage-clamp mode. The spinal cord slice was equilibrated with Krebs solution (10–15 mL/min) and irrigated with a 95% O_2_/5% CO_2_ gas mixture at 36°C ± 1°C. The Krebs solution was prepared as described in the *Immunohistochemical staining* section. The osmotic pressure of the nominally Ca^2+^-free high-Mg^2+^ (5 mM) Krebs solution was adjusted by lowering the Na^+^ concentration. After the whole-cell configuration was established, voltage-clamped neurons were held at −70 mV for recording excitatory post-synaptic currents (EPSCs) and 0 mV for recording inhibitory post-synaptic currents (IPSCs). Patch electrodes were fabricated from thin-walled borosilicate glass capillary tubes using a puller (Sutter Instrument, Novato, CA), and these exhibited a resistance of 5–10 MΩ when filled with a cesium-based intracellular solution (in mM) with the following: Cs_2_SO_4_, 110; CaCl_2_, 0.5; MgCl_2_, 2; EGTA, 5; HEPES, 5; tetraethylammonium, 5; and ATP-Mg, 5. The series resistances were assessed based on the response to a 5-mV hyperpolarizing step, and these values were continuously monitored. The data were rejected if the values changed by more than 15% during recording. The signals were amplified by filtering at 2 kHz and digitized at 5 kHz using an Axopatch 200 B amplifier (Molecular Devices). Data were collected and analyzed using the pCLAMP 10.4 software suite (Molecular Devices). We recorded miniature post-synaptic currents (mPSCs) after treatment with TTX to block the conduction of action potentials. The strength of synaptic transmission can be altered by modulating both “transmitter release probability” and “post-synaptic responsiveness,” while the analysis of the mPSCs frequency and amplitude distribution has been used to distinguish between the pre-and post-synaptic loci of experimental manipulations (Malgaroli and Tsien, 1992; Manabe et al., 1992). Moreover, presynaptic actions can affect the probability of release, and changes in the mPSC frequency indicate a presynaptic effect. By contrast, changes in mPSC amplitude can be explained by post-synaptic responsiveness alteration (Malgaroli and Tsien, 1992; Manabe et al., 1992).

Excitatory synaptic currents were evoked by stimulating the L5 dorsal root using a suction electrode. Aδ-fibers were stimulated at 100 μA (0.05 ms), and C-fibers were stimulated at 1,000 μA (0.5 ms). According to previous studies (Ohashi et al., 2017, 2022), we classified Aδ-fiber-evoked EPSCs as monosynaptic if there were no failures with repetitive stimulation at 20 Hz with short and constant latencies. We also classified C-fiber-evoked EPSCs as monosynaptic if there was no failure with repetitive stimulation at 1 Hz. Polysynaptic EPSCs were classified based on unreliable variable latencies following stimulation using the same protocols.

### 2.8 Drug administration

For the behavioral and immunohistochemical experiments and *in vivo* extracellular recording, MVIIA (Sigma-Aldrich, St. Louis, MO) or a vehicle was administered as an i.t. injection with a 10-μL bolus delivered using a catheter 4 h after contusion injury.

For the *in vitro* whole-cell, patch-clamp recordings, MVIIA and TTX (Wako, Osaka, Japan) were first dissolved in distilled water at a concentration 1,000 times greater than the final concentration for storage (MVIIA, 1 μM; TTX, 0.5 μM). Before use, we diluted these stock solutions in Krebs solution to the final concentrations, and MVIIA and TTX were bath-applied directly to the transverse spinal cord slices.

### 2.9 Statistics

Data are expressed as mean ± standard deviation (SD). To assign the rats to the experimental conditions, we did not use randomization, but adopted blinding methods during the procedures, particularly for behavioral assessments, as per previous studies (Ohashi et al., 2017, 2022). The sample sizes in this study were based on previous studies (Ohashi et al., 2017, 2022); thus, power analyses were not performed. Data obtained from the behavioral experiments were analyzed within groups. Analysis of BBB scores after i.t. injection of a vehicle or MVIIA was performed using a one-way repeated-measures analysis of variance (ANOVA). Analysis of the mechanical thresholds after i.t. injection of a vehicle or MVIIA was performed using Student’s unpaired two-tailed *t*-tests. Data obtained from the immunohistochemical studies and electrophysiological experiments were analyzed using one-way ANOVA with Bonferroni multiple *post-hoc* comparisons, Student’s paired or unpaired two-tailed *t*-tests. The Kolmogorov–Smirnov test was also performed to compare the cumulative distributions of the post-synaptic current parameters for amplitude and inter-event interval according to previous studies (Ohashi et al., 2017, 2022). StatView 5 (SAS Institute, Cary, NC, United States) was used for all statistical analyses. A *p*-value of <0.05 was considered statistically significant.

## 3 Results

### 3.1 MVIIA induces analgesia improvement of mechanical allodynia and locomotor function in SCI

**Figure 1:**
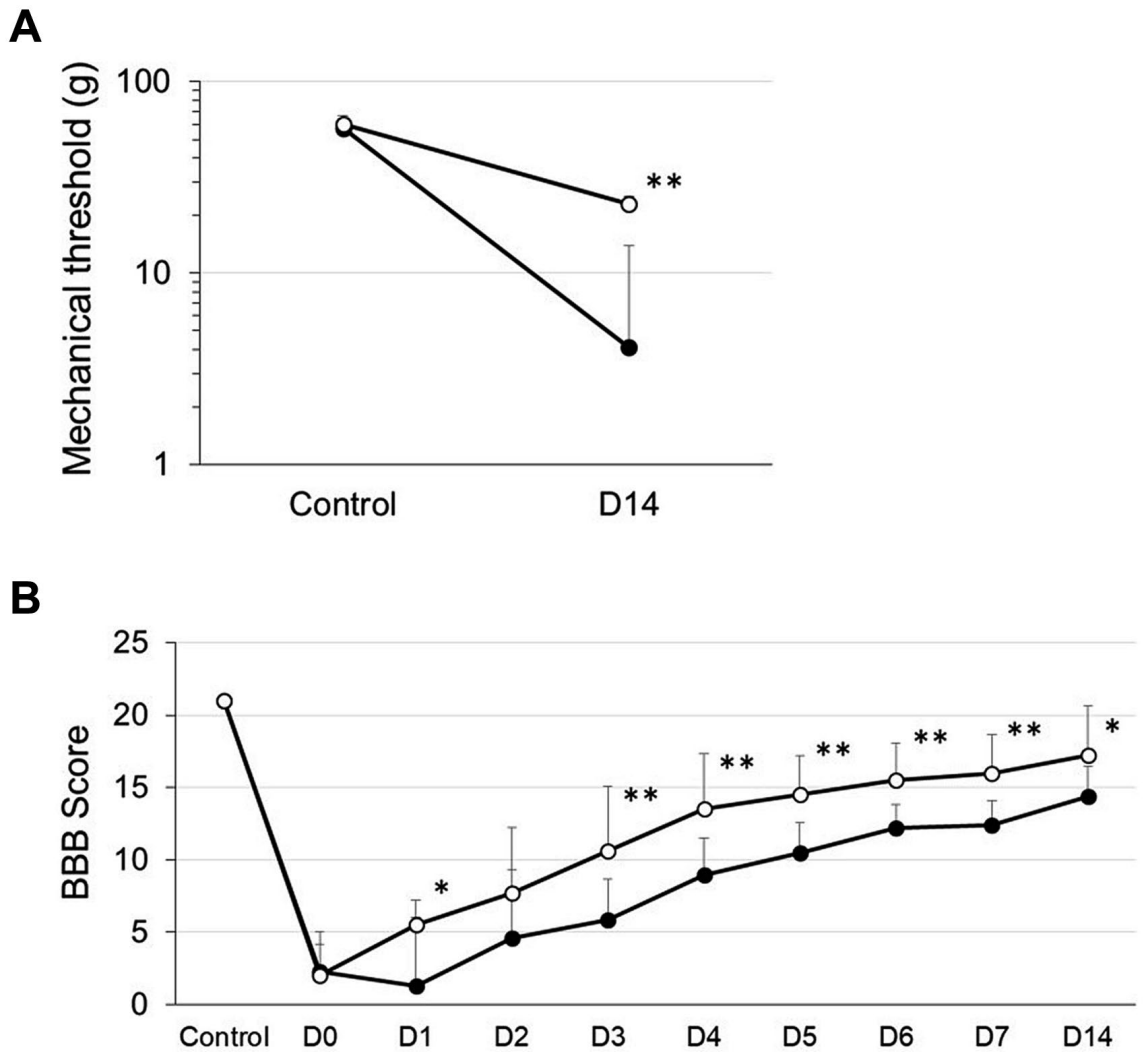
MVIIA induces analgesia improvement of mechanical allodynia and locomotor function in spinal cord injury (SCI). Rats with spinal contusion injury received a vehicle via i.t. injection 4 h after surgery (SCI rats) and received MVIIA (200 pmol) via i.t. injection 4 h after surgery (SCI + MVIIA rats). Closed circles represent SCI rats, and open circles represent SCI + MVIIA rats. **(A)** Fourteen days after contusion injury (D14), SCI + MVIIA rats revealed a significantly increased mechanical threshold for paw withdrawal compared with SCI rats (*n* = 12/SCI rats, *n* = 8/SCI + MVIIA rats). Data are presented as mean ± standard deviation (SD), ^**^p < 0.01, unpaired *t*-test. **(B)** Over the observational period of 14 days, the Basso, Beattie, and Bresnahan (BBB) score gradually increased in both SCI and SCI + MVIIA rats; however, the BBB score for SCI + MVIIA rats was significantly higher at most points than that for SCI rats (*n* = 12/SCI rats, *n* = 8/SCI + MVIIA rats). Data are presented as mean ± SD, ^*^p < 0.05, ^**^p < 0.01, one-way repeated-measures analysis of variance.

We first defined SCI rats with spinal contusion injury receiving a vehicle via i.t. injection 4 h after surgery as SCI rats and rats with spinal contusion injury receiving MVIIA (200 pmol) via i.t. injection 4 h after surgery as SCI + MVIIA rats. To assess the MVIIA analgesic action in SCI, we first investigated whether MVIIA could reduce mechanical allodynia after a contusion injury using von Frey test (Ohashi et al., 2017, 2022). At 14 days after contusion injury (day 14), SCI + MVIIA rats showed a significantly increased mechanical threshold for paw withdrawal compared with SCI rats (*n* = 12/SCI rats, 4.1 ± 2.3 g; *n* = 8/SCI + MVIIA rats; 23.0 ± 9.8 g; *p* < 0.01, unpaired *t*-test; Figure 1A). Our findings indicate that MVIIA improves locomotor function and analgesia in mechanical allodynia in SCI.

In addition to the analgesic effect and locomotor function of MVIIA in SCI, we evaluated the BBB score (Basso et al., 1995, 1996). Joint movement, paw placement, weight support, and fore/hindlimb coordination were assessed using the 21-point BBB locomotion scale. The score for the normal animals (control) was 21 points. Both SCI and SCI + MVIIA rats demonstrated a significant decrease in the BBB score on the start day of the experiment (day 0). However, the BBB score for SCI + MVIIA rats the next day (day 1) significantly increased and revealed a significant improvement in locomotor function compared with that for SCI rats (Figure 1B). Over the observation period of 14 days, the BBB score gradually increased in both groups; however, the BBB score for SCI + MVIIA rats was significantly higher at most points than that for SCI rats (*n* = 12/SCI rats, *n* = 8/SCI + MVIIA rats; *p* < 0.05, one-way repeated-measures ANOVA; Figure 1B).

### 3.2 MVIIA suppressed pERK activation in the superficial spinal dorsal horn in SCI

**Figure 2:**
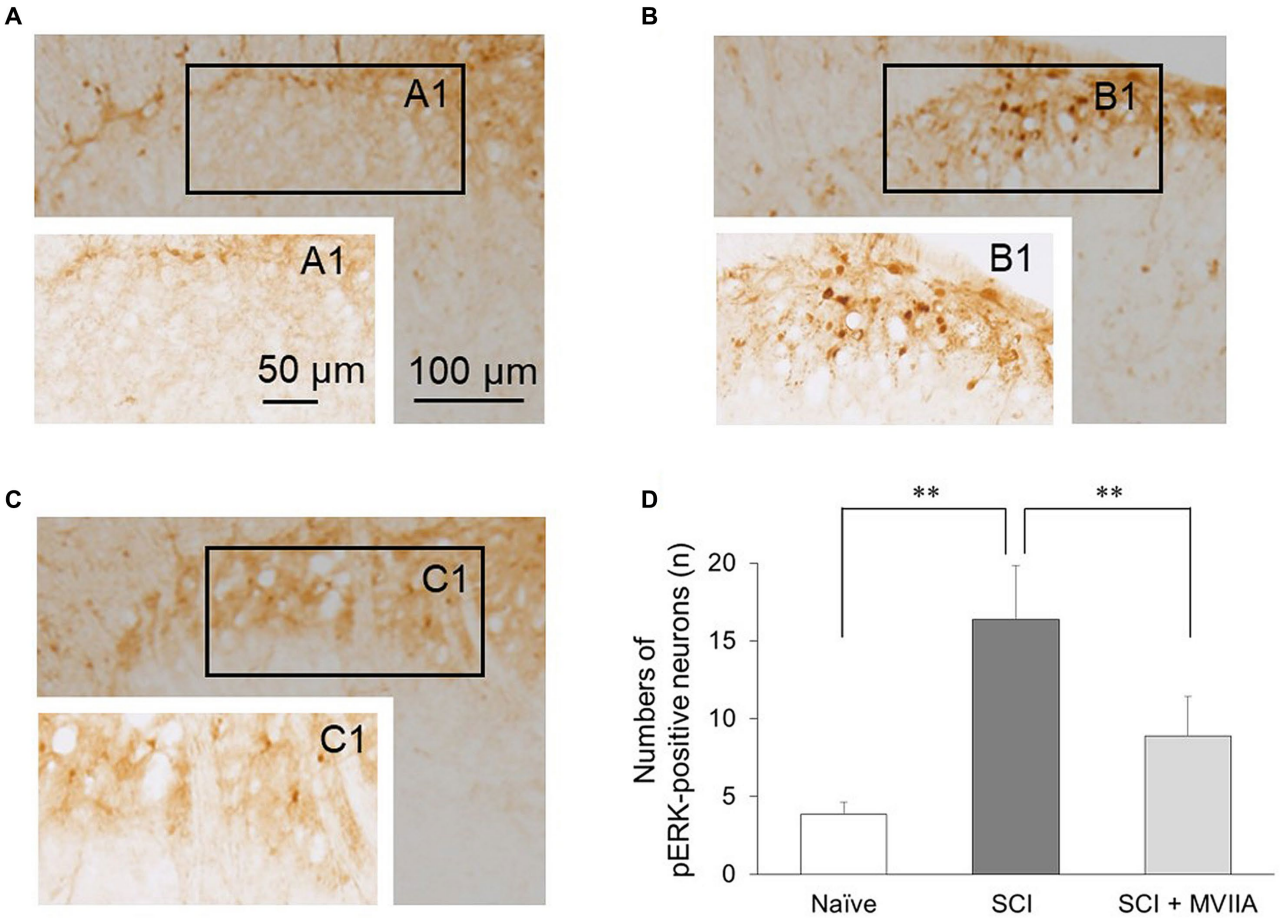
MVIIA suppresses phosphorylated extracellular signal-regulated kinase (pERK) in the spinal dorsal horn in spinal cord injury (SCI). Representative images of pERK-positive neurons in the spinal dorsal horn slices. Images corresponding to **(A)** naïve rats (*n =* 6) and **(B)** rats with spinal contusion injury receiving a vehicle by i.t. injection 4 h after surgery (*n =* 7). **(C)** Images corresponding to rats with spinal contusion injury receiving MVIIA (200 pmol) via i.t. injection 4 h after surgery (SCI + MVIIA rats, *n =* 11). **(D)** The graph represents the number of pERK-positive neurons in each slice of the spinal dorsal horn (laminae I–II). Data are presented as mean ± standard deviation, ^**^p < 0.01, one-way analysis of variance followed by Bonferroni *post-hoc* comparison.

To investigate the distribution of neurons in the superficial dorsal horn (laminae I-II) that responded to MVIIA in SCI, we examined pERK activation using immunohistochemical staining and counted the number of pERK-positive neurons in the superficial spinal dorsal horn. In the behavioral results described above, we used rats with spinal contusion injury receiving a vehicle via i.t. injection 4 h after surgery as SCI rats and rats with spinal contusion injury receiving MVIIA (200 pmol) via i.t. injection 4 h after surgery as SCI + MVIIA rats. Figures 2A,B correspond to naïve and SCI rats, respectively, and Figure 2C corresponds to SCI + MVIIA rats. The number of pERK-positive neurons in the superficial spinal dorsal horn of SCI rats (16.4 ± 3.5, *n* = 7) was significantly higher than that in the naïve rats (3.8 ± 0.8, *n* = 6; *p* < 0.01, one-way ANOVA followed by Bonferroni *post-hoc* comparison; Figure 2D). However, the number of pERK-positive neurons in the superficial spinal dorsal horn of SCI + MVIIA rats (8.9 ± 2.5, *n* = 11) was significantly lower than that in the SCI rats (*p* < 0.01, one-way ANOVA followed by Bonferroni *post-hoc* comparison; Figure 2D). These results suggest that MVIIA acts on the superficial spinal dorsal horn, suppresses the pERK-positive neurons activated by SCI, and may contribute to analgesia.

### 3.3 MVIIA acts on the superficial spinal dorsal horn directly and suppresses both spontaneous and stimulus-evoked firing in SCI

Our behavioral and immunohistochemical experiments suggest that MVIIA induces analgesia in SCI by inhibiting neuronal activity in the superficial spinal dorsal horn. Therefore, we investigated the analgesic synaptic mechanisms of SCI in the spinal dorsal horn using electrophysiology. We first investigated whether MVIIA suppressed the spinal response to both spontaneous and mechanical stimulation-evoked firing using *in vivo* extracellular recording. As described in the behavior and immunohistochemical results above, we used rats with spinal contusion injury receiving a vehicle via i.t. injection at 4 h after surgery as SCI rats and rats with spinal contusion injury receiving MVIIA (200 pmol) via i.t. injection at 4 h after surgery as SCI + MVIIA rats.

**Figure 3:**
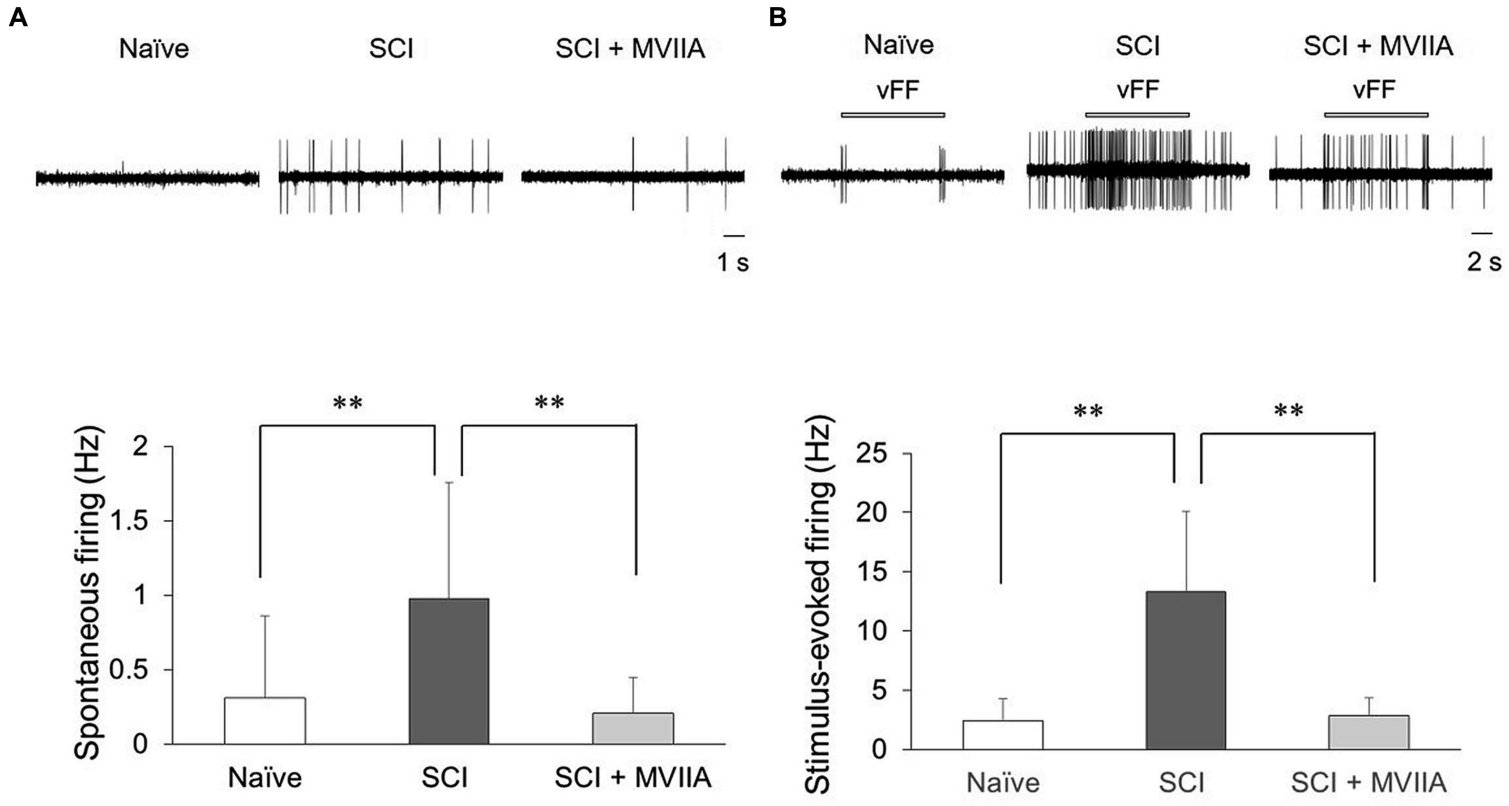
MVIIA suppresses both spontaneous and stimulus-evoked firing on the spinal dorsal horn in spinal cord injury (SCI). *In vivo* extracellular recordings of the superficial spinal dorsal horn. Rats with spinal contusion injury received a vehicle via i.t. injection 4 h after surgery (SCI rats) and received MVIIA (200 pmol) via i.t. injection 4 h after surgery (SCI + MVIIA rats). **(A)** Spontaneous firing of the superficial spinal dorsal horn in SCI + MVIIA rats (*n* = 20) was significantly lower than that in in SCI rats (*n* = 18). **(B)** Similarly, stimulus-evoked firing in the superficial spinal dorsal horn in SCI + MVIIA rats (*n* = 20) was significantly lower than that in SCI rats (*n* = 18). Data are presented as mean ± standard deviation, ^**^p < 0.01, unpaired *t*-test. vFF = von Frey Filament.

The spontaneous firing on the spinal dorsal horn in SCI rats significantly increased compared with that in naïve rats (*n* = 8/4 naïve rats, 0.31 ± 0.55 Hz; *n* = 18/4 SCI rats, 0.98 ± 0.78 Hz; *p* = 0.0081, one-way ANOVA followed by Bonferroni *post-hoc* comparison; Figure 3A). However, the spontaneous firing on the spinal dorsal horn in SCI + MVIIA rats significantly decreased compared with that in SCI rats (*n* = 20/4 SCI + MVIIA rats, 0.21 ± 0.24 Hz; *p* < 0.0010, one-way ANOVA followed by Bonferroni *post-hoc* comparison; Figure 3A).

Similarly, the stimulus-evoked firing on the spinal dorsal horn in SCI rats significantly increased compared with that in naïve rats (*n* = 8/4 naïve rats, 2.4 ± 1.9 Hz; *n* = 18/4 SCI rats, 13.3 ± 6.8 Hz; *p* < 0.0010, one-way ANOVA followed by Bonferroni *post-hoc* comparison; Figure 3B). However, the stimulus-evoked firing on the spinal dorsal horn in SCI + MVIIA rats significantly decreased compared with that in SCI rats (*n* = 20/4 SCI + MVIIA rats, 2.9 ± 1.4 Hz; *p* < 0.0010, one-way ANOVA followed by Bonferroni *post-hoc* comparison; Figure 3B). There was no difference in the recording depth of all neurons (naïve rats, 68.4 ± 35.8 mm; SCI rats, 59.6 ± 33.0 mm; SCI + MVIIA rats, 61.9 ± 34.1 mm; *p* > 0.05, one-way ANOVA followed by Bonferroni *post-hoc* comparison). These results suggest that the MVIIA acts directly on the superficial spinal dorsal horn, suppresses both spontaneous and stimulus-evoked firing activated by SCI, and may contribute to analgesia.

### 3.4 MVIIA decreases the mEPSCs frequency of substantia gelatinosa neurons in the spinal dorsal horn without changing their amplitude in SCI

Our *in vivo* extracellular recording experiments suggest that MVIIA acts directly on the superficial spinal dorsal horn. Therefore, we investigated the analgesic mechanism of the MVIIA in the dorsal horn of the spine. In the superficial spinal dorsal horn, substantia gelatinosa neurons in lamina II contain many types of neurotransmitters and express various receptors and channels that modulate nociceptive information. Furthermore, N-type voltage-dependent calcium channels are reportedly expressed in lamina II of the spinal dorsal horn, and the expression of N-type voltage-dependent calcium channels is augmented in neuropathic pain (Takasu et al., 2016). Therefore, we investigated the effects of MVIIA on synaptic transmission in substantia gelatinosa neurons in lamina II with *in vitro* patch-clamp recording using rats with spinal contusion injury as SCI rats.

**Figure 4:**
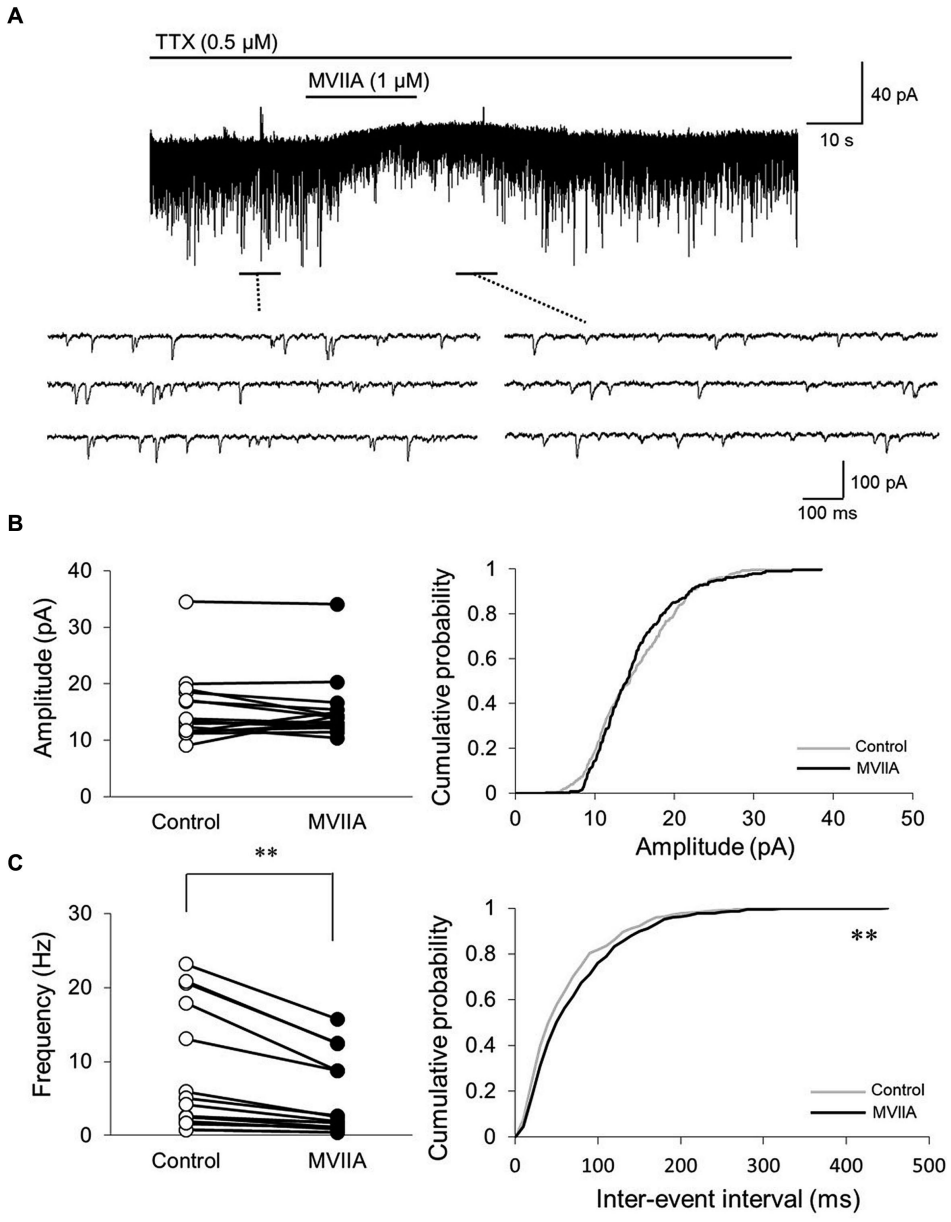
MVIIA inhibits the presynaptic excitatory interneurons of substantia gelatinosa neurons in the spinal dorsal horn in spinal cord injury (SCI). *In vitro* patch-clamp recordings of substantia gelatinosa neurons in the spinal dorsal of SCI rats with spinal contusion injury. **(A)** Miniature excitatory post-synaptic currents (mEPSCs) were isolated by adding tetrodotoxin (TTX, 0.5 μM) and direct application of MVIIA to the spinal cord (1 μM, 30 s) in SCI rats. **(B)** Direct application of MVIIA to the spinal cord did not change the mean mEPSCs amplitude (*n* = 16; paired *t*-test) or affect the cumulative distribution of mEPSCs amplitudes (Kolmogorov–Smirnov test). **(C)** MVIIA significantly decreased the mean mEPSCs frequency (*n* = 16; paired *t*-test), and the cumulative inter-event interval distribution showed a significant rightward shift (Kolmogorov–Smirnov test). Data are presented as mean ± standard deviation, ^**^p < 0.01. TTX = tetrodotoxin.

We investigated whether MVIIA affected excitatory synaptic transmission in substantia gelatinosa neurons. The mEPSCs were isolated by adding tetrodotoxin (TTX, 0.5 μM). While direct MVIIA application to the spinal cord (1 μM, 20 s) in SCI rats did not affect the mean mEPSCs amplitude (control, 15.4 ± 6.0 pA; MVIIA, 15.1 ± 5.6 pA; 101.0% ± 20.3% of control; *n* = 16; *p* = 0.62, paired *t-*test; Figures 4A,B), it significantly decreased the mean mEPSCs frequency under MVIIA (control, 7.8 ± 8.2 Hz; MVIIA, 4.6 ± 5.2 Hz; 57.5% ± 10.2% of control; *n* = 16; *p* = 0.0020, paired *t-*test; Figures 4A,C). MVIIA elicited outward currents in 5 of 16 cells (6.1 ± 1.6 pA). MVIIA did not affect the cumulative distribution of mEPSCs amplitudes; however, the cumulative inter-event interval distribution of mEPSCs demonstrated a significant rightward shift under MVIIA relative to the control (Kolmogorov–Smirnov test, *p* < 0.01; Figures 4B,C). Considering that changes in the mEPSCs frequency reflect presynaptic glutamate release, our results suggest that MVIIA inhibits presynaptic glutamate release from substantia gelatinosa neurons in SCI rats.

### 3.5 MVIIA does not affect the mIPSCs of substantia gelatinosa neurons in the spinal dorsal horn in SCI

**Figure 5:**
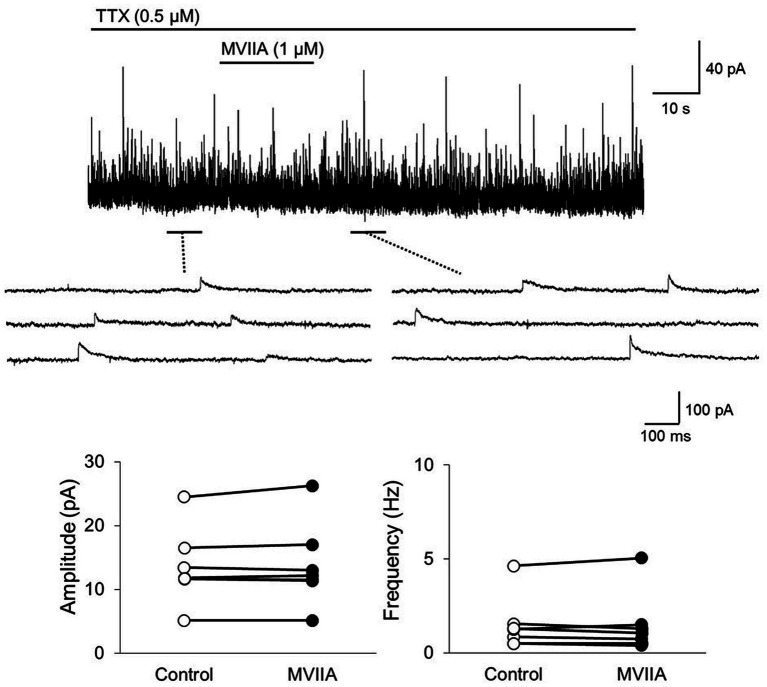
MVIIA does not affect the inhibitory interneurons of substantia gelatinosa neurons in the spinal dorsal horn in spinal cord injury (SCI). *In vitro* patch-clamp recordings of substantia gelatinosa neurons in the spinal dorsal of SCI rats with spinal contusion injury. Miniature inhibitory post-synaptic currents (mIPSCs) were isolated by adding tetrodotoxin (TTX, 0.5 μM) and direct application of MVIIA to the spinal cord (1 μM, 30 s) in SCI rats. The direct application of MVIIA to the spinal cord did not change the mean mIPSCs amplitude (*n* = 7; paired *t*-test) or frequency (*n* = 7; paired *t*-test). Data are presented as mean ± standard deviation. TTX = tetrodotoxin.

Subsequently, we investigated whether MVIIA affected inhibitory synaptic transmission in substantia gelatinosa neurons in lamina II with *in vitro* patch-clamp recording using rats with spinal contusion injury as SCI rats. mIPSCs were isolated by adding TTX (0.5 μM). Direct MVIIA application (1 μM, 20 s) in SCI rats did not affect the mean mIPSCs amplitude (control, 13.5 ± 5.9 pA; MVIIA, 13.8 ± 6.5 pA; 101.1% ± 3.7% of control; *n* = 7; *p* = 0.38, paired *t*-test; Figure 5) or frequency (control, 1.5 ± 1.4 Hz; MVIIA, 1.5 ± 1.6 Hz, 94.7% ± 15.3% of control; *n* = 7; *p* = 0.96, paired *t*-test; Figure 5). These results suggest that MVIIA does not affect inhibitory interneurons in substantia gelatinosa neurons in SCI rats.

### 3.6 MVIIA reduces the amplitudes of monosynaptic Aδ-and C-fiber-evoked EPSCs on substantia gelatinosa neurons in the spinal dorsal horn in SCI

Our behavioral evidence and *in vivo* extracellular recording experiments suggest that MVIIA inhibits neuronal activity evoked by mechanical stimulation. N-type voltage-dependent calcium channels are reportedly expressed on primary afferent fibers of the spinal dorsal horn, and the expression of N-type voltage-dependent calcium channels is augmented in neuropathic pain (Takasu et al., 2016). Therefore, we examined MVIIA effects on EPSCs in lamina II evoked by the stimulation of primary afferent Aδ-and C-fibers via dorsal roots with *in vitro* patch-clamp recording using rats with spinal contusion injury as SCI rats.

**Figure 6:**
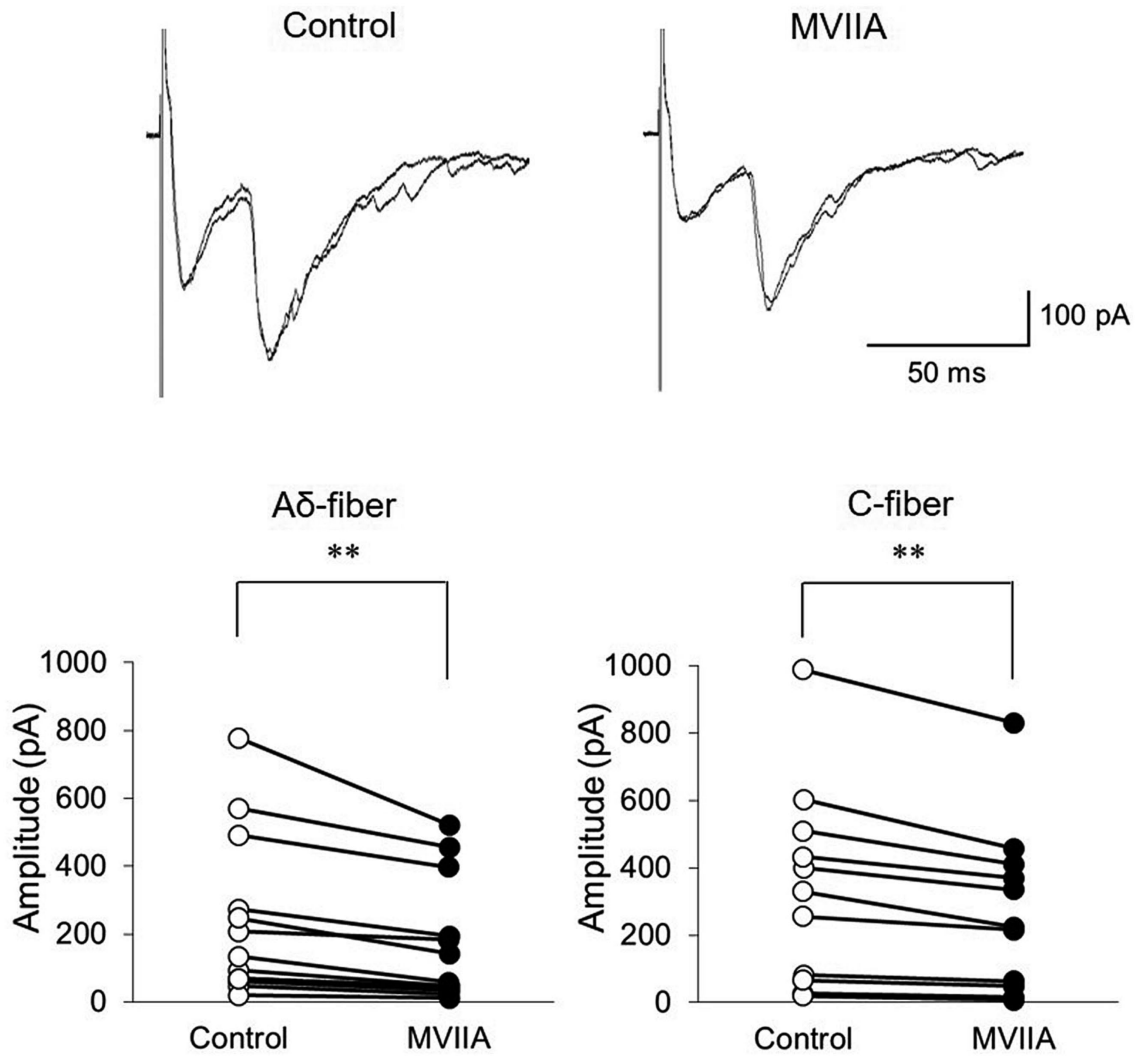
MVIIA inhibits the amplitude of monosynaptic glutamatergic evoked-excitatory post-synaptic currents (EPSCs) expressed on primary afferent Aδ-and C-fibers of substantia gelatinosa neurons in the spinal dorsal horn in spinal cord injury (SCI). *In vitro* patch-clamp recordings of substantia gelatinosa neurons in the spinal dorsal of SCI rats with spinal contusion injury. Direct application of MVIIA (1 μM, 30 s) decreased both the amplitudes of monosynaptic Aδ-fiber-evoked EPSCs (*n* = 15; paired *t-*test) and those of monosynaptic C-fiber-evoked EPSCs (*n* = 11; paired *t-*test). Data are presented as mean ± standard deviation, ^**^p < 0.01.

Direct MVIIA application (1 μM, 20 s) in SCI rats decreased both the amplitudes of monosynaptic Aδ-fiber-evoked EPSCs (control, 212.7 ± 227.3 pA; MVIIA, 149.5 ± 171.1 pA; 64.4% ± 12.6% of control; *n* = 15; *p* = 0.0018, paired *t-*test; Figure 6) and those of monosynaptic C-fiber-evoked EPSCs (control, 337.8 ± 296.5 pA; MVIIA, 270.9 ± 247.8 pA; 74.4% ± 11.7% of control; *n* = 11; *p* = 0.0020, paired *t-*test; Figure 6). These findings indicate that MVIIA reduces the amplitudes of monosynaptic Aδ-and C-fiber-evoked EPSCs on substantia gelatinosa neurons in SCI rats.

### 3.7 MVIIA does not affect mEPSCs frequency and amplitudes of monosynaptic Aδ-and C-fiber-evoked EPSCs on substantia gelatinosa neurons in SCI under Ca2+ free circumstances

**Figure 7:**
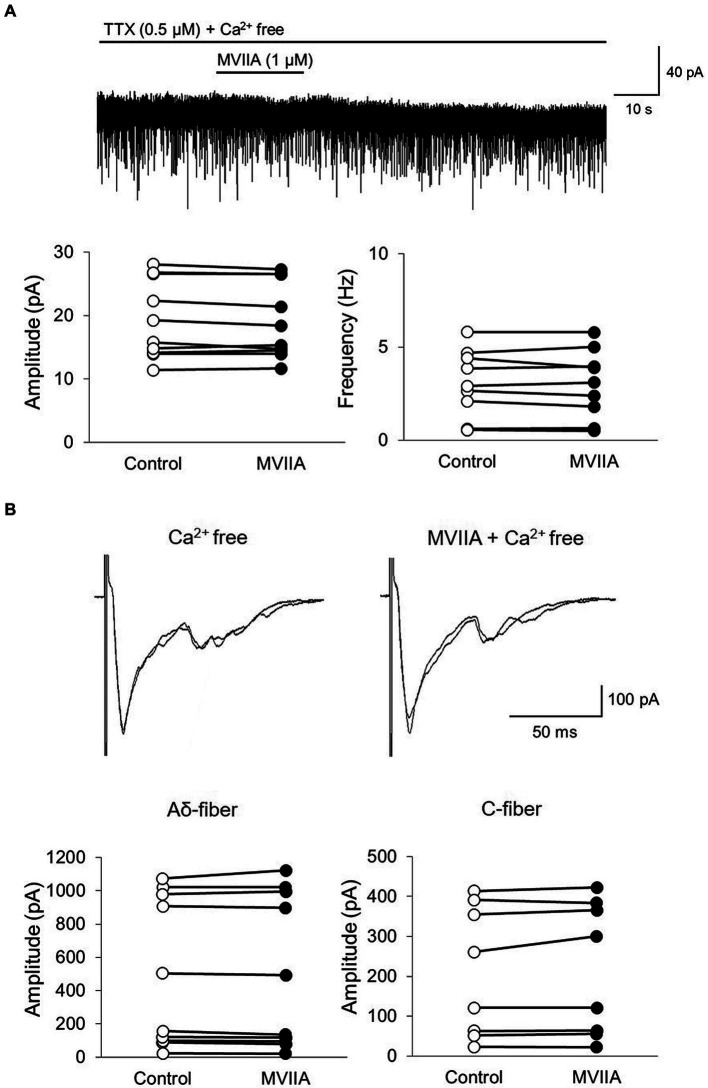
MVIIA does not affect the miniature excitatory post-synaptic currents (mEPSCs) frequency and amplitudes of monosynaptic glutamatergic evoked EPSCs expressed on primary afferent Aδ-and C-fibers of substantia gelatinosa neurons in the spinal dorsal horn in spinal cord injury (SCI) under Ca^2+^ free circumstances. *In vitro* patch-clamp recordings of substantia gelatinosa neurons in the spinal dorsal of spinal cord injury rats with spinal contusion injury. **(A)** In Ca^2+^ free Krebs, direct application of MVIIA (1 μM, 30 s) does not change the mean mEPSCs amplitude (*n* = 10; paired *t*-test) or frequency (*n* = 10; paired *t*-test). Data are presented as mean ± standard deviation. TTX = tetrodotoxin. **(B)** In Ca^2+^ free Krebs, direct application of MVIIA (1 μM, 30 s) does not change the amplitudes of monosynaptic Aδ-fiber-evoked EPSCs (*n* = 10; paired *t-*test) or those of monosynaptic C-fiber-evoked EPSCs (*n* = 8; paired *t-*test). Data are presented as mean ± standard deviation.

MVIIA is an N-type voltage-dependent calcium-channel blocker; thus, we examined whether MVIIA-induced decreases in mEPSCs frequency and amplitudes of monosynaptic Aδ-and C-fiber-evoked EPSCs were dependent on Ca^2+^. In Ca^2+^ free Krebs, direct MVIIA application (1 μM, 20 s) in SCI rats did not affect the mean mEPSCs amplitude (control, 19.3 ± 6.2 pA; MVIIA, 19.0 ± 6.0 pA, 98.9% ± 3.2% of control; *n* = 10; *p* = 0.15, paired *t*-test; Figure 7A) or frequency (control, 2.8 ± 1.9 Hz; MVIIA, 2.8 ± 1.9 Hz, 98.0% ± 8.4% of control; *n* = 10; *p* = 0.57, paired *t*-test; Figure 7A). Similarly, in Ca^2+^ free Krebs, direct MVIIA application (1 μM, 20 s) in SCI rats did not affect the amplitudes of monosynaptic Aδ-fiber-evoked EPSCs (control, 497.1 ± 449.0 pA; MVIIA, 498.2 ± 461.0 pA; 96.5% ± 5.8% of control; *n* = 10; *p* = 0.86, paired *t*-test; Figure 7B) or the amplitudes of monosynaptic C-fiber-evoked EPSCs (control, 210.1 ± 163.6 pA; MVIIA, 217.2 ± 167.6 pA; 102.7% ± 6.1% of control; *n* = 8; *p* = 0.20, paired *t*-test; Figure 7B). These findings indicate that MVIIA does not affect mEPSCs frequency and amplitudes in monosynaptic Aδ-and C-fiber-evoked EPSCs on substantia gelatinosa neurons in SCI rats under Ca^2+^ free conditions.

## 4 Discussion

Patients with SCI experience motor dysfunction and neuropathic pain. In particular, neuropathic pain caused by SCI gradually worsens and directly aggravates quality of life (Mariano, 1992; Yezierski, 1996; Siddall et al., 1999, 2003; Hulsebosch, 2002; Ackery et al., 2004; Rossignol et al., 2007; Oyinbo, 2011). Causes of SCI-induced pain include necrosis, apoptosis, oxidative stress, excitotoxicity, and neuronal tissue degeneration in the spinal cord (Choi, 1992; Isaac and Pejic, 1995; Yezierski, 1996; Amar and Levy, 1999; Schumacher et al., 1999; Carlson and Gorden, 2002; Hall and Springer, 2004; Kwon et al., 2004; Aslan et al., 2009; Torres et al., 2010; Liu et al., 2011; Oyinbo, 2011; Hall et al., 2016; Xia et al., 2017). Therefore, treatments for SCI-induced pain have been based on central and peripheral neuropathic pain treatments, such as amitriptyline, gabapentin, pregabalin, and tramadol (Siddall and Loeser, 2001; Finnerup, 2013; Siddall and Middleton, 2015). However, causes of SCI-induced pain are very complex, and the precise cellular mechanisms remain unknown; therefore, these medications have minimal effect on SCI-induced pain, and many patients continue to suffer for an extended period (Mariano, 1992; Yezierski, 1996; Siddall et al., 1999, 2003; Hulsebosch, 2002; Ackery et al., 2004; Rossignol et al., 2007; Oyinbo, 2011). SCI-induced pain was recently revealed to be caused by excessive intracellular calcium accumulation and voltage-gated calcium-channel upregulation, leading to critical steps in ionic dysregulation (Choi, 1992; Isaac and Pejic, 1995; Amar and Levy, 1999; Schumacher et al., 1999; Hall and Springer, 2004; Aslan et al., 2009; Torres et al., 2010; Liu et al., 2011; Oyinbo, 2011). Calcium-channel blockers have neuroprotective effects against SCI by preventing the intense influx of calcium ions and, consequently, secondary injury progression (Choi, 1992; Isaac and Pejic, 1995; Amar and Levy, 1999; Aslan et al., 2009; Torres et al., 2010; Liu et al., 2011; Oyinbo, 2011). SCI model rats with contusion injury exhibited upregulation or a higher expression of voltage-gated calcium channels in the spinal cord (Boroujerdi et al., 2011; Kusuyama et al., 2018; Bhattacharyya et al., 2020). N-type voltage-dependent calcium channels are highly expressed in the spinal dorsal horn, where they modulate nociceptive transmission (Gohil et al., 1994; Westenbroek et al., 1998). Therefore, we considered that N-type voltage-dependent calcium-channel blockers may improve SCI-induced pain. Although other neuropathic pain models, such as the spinal-nerve ligation model, have shown pain reduction after N-type voltage-dependent calcium-channel blocker administration (Chaplan et al., 1994; Bowersox et al., 1996; Chang et al., 2015), few studies have investigated the mechanism of N-type voltage-dependent calcium-channel blockers in SCI-induced pain. MVIIA is the only calcium-channel blocker clinically approved as an analgesic drug for treating severe chronic pain (Bingham et al., 2010; Lewis et al., 2012; Sanford, 2013; McDowell and Pope, 2016). Recently, a human clinical study using ziconotide, N-type voltage-dependent calcium-channel blockers: MVIIA, in patients with SCI-induced pain reported a decrease in pain (Brinzeu et al., 2019); however, the effects and mechanisms of MVIIA on SCI-induced pain are still fully unknown. For example, a behavioral study using animal models investigated the effect of MVIIA administration 3 weeks after SCI on neuropathic pain (Hama and Sagen, 2009), while another study investigated the cell viability, including mitochondrial viability or cell death value (Oliveira et al., 2018). Therefore, the direct spinal analgesic effect and spinal cellular mechanisms of MVIIA were not investigated, especially when administered in the acute phase after SCI, on neuropathic pain. Therefore, we assessed the analgesic mechanisms of MVIIA by examining its effects on synaptic transmission in the spinal dorsal horn using behavioral and immunohistochemical analyses and electrophysiological experiments. We revealed that MVIIA administration in the acute phase after SCI induces analgesia against SCI-induced pain, and these analgesic mechanisms may be caused by the action of MVIIA inhibiting presynaptic N-type voltage-dependent calcium-channel blockers expressed on primary afferent Aδ-and C-fibers of substantia gelatinosa neurons in the spinal dorsal horn.

Herein, to assess the neuroprotective effects of MVIIA in SCI, we investigated the effects on analgesia and motor function recovery. Many studies have been conducted to promote the neuroprotective effects of MVIIA on motor function. Steroids were the only drugs evaluated in Phase III trials. The use of corticosteroids in patients with SCI began in the 1960s, hypothesizing that corticosteroids with anti-inflammatory properties would also reduce spinal cord edema, based on the experience of using steroids for brain swelling. Many studies support the positive effects of steroid use in SCI; however, in the early 2000s, the occurrence of complications with high-dose steroids in SCI was continuously reported. Further, the effectiveness of steroids in SCI was unclear (Bracken et al., 1990; Fehlings et al., 2017; Lee and Jeong, 2022). Therefore, the use of steroids for SCI is not necessarily recommended, and there is no established method to improve motor function. In our study, we discovered that MVIIA improved analgesia for mechanical allodynia and locomotor function in SCI. Therefore, our study suggests that MVIIA administration in the acute phase after SCI may be a novel treatment strategy for targeting neuropathic pain and motor function.

Analgesic studies have demonstrated that MVIIA concentrations of 3–200 μM effectively block N-type voltage-dependent calcium channels (Oliveira et al., 2018). The incidence of side effects limited the use of higher MVIIA concentrations; side effects become more intense and more frequent at higher MVIIA doses (Souza et al., 2008; Oliveira et al., 2018). Doses greater than the maximum recommended dose exaggerate pharmacological effects (ataxia, nystagmus, dizziness, stupor, unresponsiveness, spinal myoclonus, confusion, sedation, hypotension, word-finding difficulties, garbled speech, nausea, and vomiting); however, Hama and Sagen reported that MVIIA at a dose of 5 μM did not cause complications (Hama and Sagen, 2009). Nevertheless, due to safety reasons, clinical use of MVIIA is limited to low doses. Generally, the recommended clinical maximum i.t. MVIIA dose is 19.2 μg/day, although the maximum i.t. MVIIA dose administered in clinical trials was 912 μg/day (Smith and Deer, 2009). MVIIA pharmacokinetics in the cerebrospinal fluid have been studied after an i.t. MVIIA dose of 1–10 μg in patients with chronic pain. Following i.t. administration of 1–10 μg of MVIIA, total exposure (area under the receiver operating characteristics curve: 83.6–608 ng·h/mL) and peak exposure (Cmax range: 16.4–132 ng/mL) values in the cerebrospinal fluid were variable-and dose-dependent but appeared approximately dose-proportional (Wermeling et al., 2003; Wermeling, 2005). Therefore, the MVIIA concentration that demonstrated an analgesic effect in previous animal studies was higher than the clinical dose. However, other studies reported that lower MVIIA doses are sufficient to elicit N-type voltage-dependent calcium-channel inhibition (Souza et al., 2008; de Souza et al., 2011). In the current study, to use a dosage as close to the clinical dosage as possible, we chose a relatively lower dose than those used in previous animal studies: 200 pmol in behavioral and immunohistochemical experiments and *in vivo* extracellular recording, and 1 μM in *in vitro* patch-clamp recording.

According to Valentino et al. and Verweij et al., MVIIA injections 15 min before or 1 h after ischemic or traumatic brain injuries, respectively, do not reveal a neuroprotective effect (Valentino et al., 1993; Verweij et al., 2000). By contrast, Oliveira et al. reported that MVIIA administration 4 h after SCI enhanced mitochondrial and cell viability (Oliveira et al., 2018). We also demonstrated the positive effects of MVIIA administration 4 h after SCI on both neuropathic pain and motor function recovery. The reason for the discrepancy in the neuroprotective effects due to the timing of MVIIA administration remains unknown. Verweij et al. stated that MVIIA should be more effective during critical calcium periods (Verweij et al., 2000). Since calcium levels peak 8 h after SCI (Happel et al., 1981), MVIIA injection 4 h after trauma should have a maximum effect between 3 h and 4 h after its application, coinciding with the peak intracellular calcium concentration (de Souza et al., 2011, 2013). These results suggest that MVIIA injection 4 h after SCI exerts a neuroprotective effect by inhibiting calcium elevation. Therefore, we administered i.t. MVIIA 4 h after contusion injury for behavioral and immunohistochemical experiments and *in vivo* extracellular recording. By contrast, we selected a method to directly administer MVIIA to the transverse spinal cord slices of SCI rats 14 days after contusion injury in our *in vitro* patch-clamp recording; however, it remains unclear whether the action of MVIIA observed in the *in vitro* experiments occurred due to the mechanisms of the analgesic effects on SCI + MVIIA rats with spinal contusion injury receiving MVIIA via i.t. injection 4 h after surgery. As the characteristics of the reactions that occur in each cell of the *in vitro* patch-clamp recording vary substantially, we could not directly compare the spinal responses between the SCI and SCI + MVIIA rats. Furthermore, neuropathic pain causes plasticity in the brain and spinal cord, and SCI also causes central neuropathic pain and modulates the pain pathway (Boadas-Vaello et al., 2016; Vierck, 2020; Guérout, 2021). Neuroimaging studies have reported that SCI leads to alterations in brain structure and brain function in patients (Solstrand Dahlberg et al., 2018). Moreover, SCI causes central neuropathic pain by affecting the descending inhibitory pathway, leading to hypofunction of γ-aminobutyric acid (GABA)-mediated modulation in the spinal dorsal horn (Gwak and Hulsebosch, 2011). These responses suggest that GABA receptors on the spinal dorsal horn are affected by brain and spinal plasticity resulting from SCI-induced pain. SCI also induces spinal microglial cell reactivation, which leads to an increase in the levels of inflammatory mediators and hyperexcitability of the spinal nociceptive projection neurons and afferent primary nerve fibers at the spinal dorsal horn located rostral and caudal to the lesion site (Boadas-Vaello et al., 2016). Therefore, it was difficult to distinguish whether the spinal responses using *in vitro* patch-clamp recording were reflected by MVIIA via i.t. injection 4 h after spinal contusion injury. Hence, we opted to directly administer MVIIA to the transverse spinal cord slices from SCI rats in our *in vitro* patch-clamp recording to exclude these factors and to evaluate the local action of MVIIA on spinal dorsal horn under SCI-induced pain.

**Figure 8:**
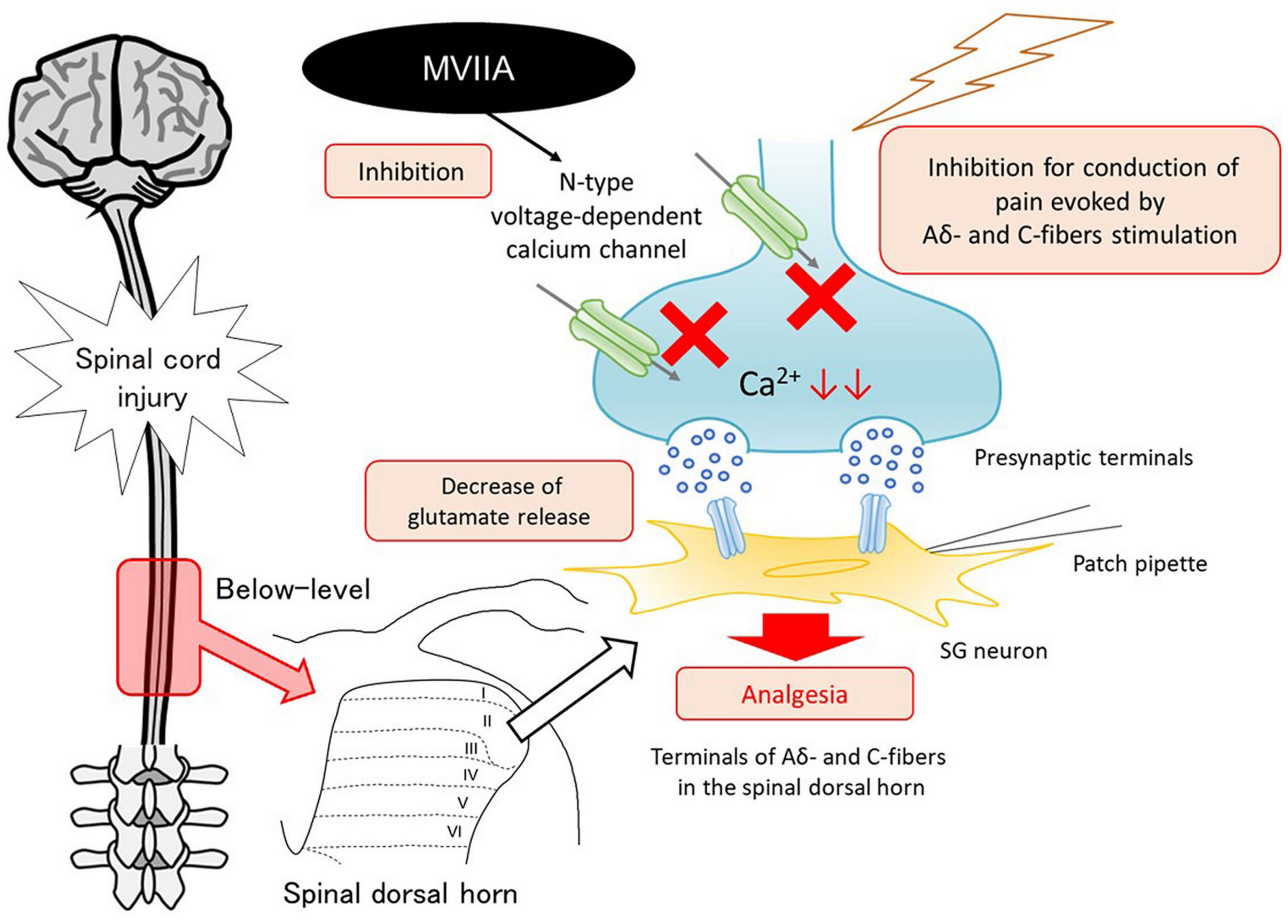
Mechanism of analgesic action of MVIIA in the spinal dorsal horn in spinal cord injury (SCI). MVIIA acts on N-type voltage-dependent calcium channels on both Aδ-and C-fiber terminals in the spinal dorsal horn to inhibit the conduction of pain evoked by stimulation and decrease glutamate release. These mechanisms suggest that administration of MVIIA in the acute phase after SCI may contribute to the analgesic effect, inhibiting spinal neuronal excitability enhanced by SCI-induced pain.

Voltage-gated calcium channels are divided into L, N, P/Q, R, and T types based on their structural and pharmacological properties. Different types of calcium channels exhibit different cellular and subcellular distributions. N-type voltage-dependent calcium channels are expressed at presynaptic central or peripheral terminals of neurons, where they control the calcium-dependent release of neurotransmitters to initiate synaptic transmission (Gohil et al., 1994; Olivera et al., 1994). N-type voltage-dependent calcium channels are also expressed at a high density on the terminals of primary afferent neurons in the spinal dorsal horn (Gohil et al., 1994; Olivera et al., 1994). This area is important for pain modulation and processing. Once nociceptors on the neurons in the spinal dorsal horn are activated via Aδ and C nociceptive afferent fiber stimulation, excitatory neurotransmitters such as glutamate and neuropeptides are released, and pain information is modulated and conveyed to supraspinal brain areas. Thus, the spinal dorsal horn is an important site for nociceptive transmission. MVIIA binds with high affinity to N-type voltage-dependent calcium channels and acts as a selective N-type voltage-dependent calcium-channel blocker; therefore, after binding to N-type voltage-dependent calcium channels in the spinal dorsal horn, calcium influx into the nerve terminals is blocked, thereby reducing the release of excitatory neurotransmitters, mainly glutamate, from the primary afferent nerve terminals into the synaptic cleft. Furthermore, it is reported that the expression of N-type voltage-dependent calcium channels is upregulated in the spinal dorsal horn after nerve injury (Takasu et al., 2016), and N-type voltage-dependent calcium channels are essential in nociceptive processing under pathological conditions (Cizkova et al., 2002; Yokoyama et al., 2003). These findings suggest that this process may contribute to the spinal analgesic effects of MVIIA. Our behavioral and immunohistochemical studies revealed that i.t. MVIIA injections improved analgesia in SCI-induced pain. Considering that changes in the mEPSCs frequency reflect presynaptic glutamate release, we demonstrated in our electrophysiological study that these phenomena are caused by MVIIA acting on the spinal dorsal horn directly, which showed that MVIIA decreases mEPSCs frequency and reduces the amplitudes of monosynaptic Aδ-and C-fiber-evoked EPSCs. Thus, MVIIA inhibits presynaptic glutamate release from the substantia gelatinosa neurons under SCI-induced pain. We propose a model circuit for the analgesic mechanism of MVIIA in the spinal dorsal horn in SCI-induced pain (Figure 8).

Our study has some limitations. First, we could not explore the spinal mechanism of recovery of motor function in detail. The synaptic methods using electrophysiological experiments to investigate the spinal motor function have not been established; thus, further studies investigating the spinal mechanism of MVIIA in the recovery of motor function against SCI are needed. Second, our study only focused on the acute phase after SCI. However, many studies reported that neuropathic pain related to SCI developed over time and a large proportion of SCI patients continue to experience neuropathic pain many years after SCI (Finnerup et al., 2014, 2016; Brinzeu et al., 2019). Therefore, our study using MVIIA in the acute phase after SCI injury is less likely to be clinically useful for SCI neuropathic pain, which develops over a period of time in SCI patients and the treatment for SCI-induced pain may not be sought in the earlier acute phase following SCI injury. Indeed, a study recently reported that the late presenting SCI-induced pain resulted in more intense painful electrical and cold sensations compared to the SCI-induced pain observed in the acute phase after injury (Rosner et al., 2022). Phenotypic differences between acute and late presenting SCI-induced pain support the incorporation of timing into a mechanism-based classification of SCI-induced pain, and a delayed mechanism for the SCI-induced pain suggests the presence of a window of opportunity to prevent pain development after SCI, which may be relevant to clinical trial design and broaden our understanding of mechanisms in SCI-induced pain.

In conclusion, we revealed that MVIIA administration in the acute phase after SCI induces analgesia in SCI-induced pain, and these analgesic mechanisms may be caused by the action of MVIIA inhibiting N-type voltage-dependent calcium channels on both Aδ-and C-fiber terminals in the spinal dorsal horn, resulting in a decrease in neuronal excitability enhanced by SCI-induced pain. Our data could be useful for the clinical management of SCI-induced pain with MVIIA.

## Data availability statement

The original contributions presented in the study are included in the article/supplementary material, further inquiries can be directed to the corresponding author.

## Ethics statement

The animal study was approved by Institutional Animal Care and Use Committee of the Niigata University Graduate School of Medical and Dental Science (approval no. SA01015). The study was conducted in accordance with the local legislation and institutional requirements.

## Author contributions

NO: Conceptualization, Data curation, Formal analysis, Funding acquisition, Investigation, Methodology, Project administration, Resources, Software, Supervision, Validation, Visualization, Writing – original draft. DU: Data curation, Investigation, Writing – original draft. MO: Data curation, Investigation, Writing – original draft. RH: Data curation, Investigation, Writing – original draft. HB: Supervision, Writing – original draft.
